# Supplementary material for: Influence of parental involvement and parenting styles in children’s active lifestyle: a systematic review
Source: PeerJ. 2023 Dec 21;11:e16668. doi: 10.7717/peerj.16668 (PMC10749091; doi:10.7717/peerj.16668)
Supplement: Supplemental Information 1 [file peerj-11-16668-s001.docx]

**Appendix**

*PRISMA Checklist*

| **Section/Topic** | **Ítem nº** | **checklist item** | **Reported on page nº** |
| --- | --- | --- | --- |
| *Títle*  Títle | 1 | Identify the report as a systematic review. |  |
| *Abstract*  ***Title***  ***Background.*** *Objectives*  ***Methods***  *Eligibility criterian*  *Information sources*  *Risk of bias*  *Synthesis of results*  ***Results***  *Included studies*  Synthesis of result  **Discussion**  Limitation of evidence  Interpretation  **Other**  Funding  Registration | 2 | 1. Identify the report as a systematic review. 2. Provide an explicit statement of the main objective(s) or question(s) the review addresses. 3. Specify the inclusion and exclusion criteria for the review. 4. Specify the information sources (e.g. databases, registers) used to identify studies and the date when each was last searched. 5. Specify the methods used to assess risk of bias in the included studies. 6. Specify the methods used to present and synthesise results. 7. Give the total number of included studies and participants and summarise relevant characteristics of studies 8. Present results for main outcomes, preferably indicating the number of included studies and participants for each. If meta-analysis was done, report the summary estimate and confidence/credible interval. If comparing groups, indicate the direction of the effect (i.e. which group is favoured) 9. Provide a brief summary of the limitations of the evidence included in the review (e.g. study risk of bias, inconsistency and imprecision). 10. Provide a general interpretation of the results and important implications 11. Specify the primary source of funding for the review 12. Provide the register name and registration number. | 2  2  -  2  -  -  2  2  2  2  2  -  - |
| *Introduction* Rationale  Objectives |  | 3. Describe the rationale for the review in the context of existing knowledge.  4. Provide an explicit statement of the objective(s) or question(s) the review addresses. | 3  3, 5, 6 |
| ***Methods***  Eligibility criteria  Information sources  Search strategy  Selection process  Data collection process  Data ítems  Study risk of bias assessment  Effect measures  Synthesis methods  Reporting bias assessment  Certainty assessment |  | 5. Specify the inclusion and exclusion criteria for the review and how studies were grouped for the syntheses  6. Specify all databases, registers, websites, organisations, reference lists and other sources searched or consulted to identify studies. Specify the date when each source was last searched or consulted.  7. Present the full search strategies for all databases, registers and websites, including any filters and limits used.  8. Specify the methods used to decide whether a study met the inclusion criteria of the review, including how many reviewers screened each record and each report retrieved, whether they worked independently, and if applicable, details of automation tools used in the process.  9. Specify the methods used to collect data from reports, including how many reviewers collected data from each report, whether they worked independently, any processes for obtaining or confirming data from study investigators, and if applicable, details of automation tools used in the process  10 a. List and define all outcomes for which data were sought. Specify whether all results that were compatible with each outcome domain in each study were sought (e.g. for all measures, time points, analyses), and if not, the methods used to decide which results to collect.  10 b. List and define all other variables for which data were sought (e.g. participant and intervention characteristics, funding sources). Describe any assumptions made about any missing or unclear information  11. Specify the methods used to assess risk of bias in the included studies, including details of the tool(s) used, how many reviewers assessed each study and whether they worked independently, and if applicable, details of automation tools used in the process.  12.Specify for each outcome the effect measure(s) (e.g., risk ratio, mean difference) used in the synthesis or presentation of the results  13a Describe the processes used to decide which studies were eligible for each synthesis (e.g. tabulating the study intervention characteristics and comparing against the planned groups for each synthesis (item #5))  13b Describe any methods required to prepare the data for presentation or synthesis, such as handling of missing summary statistics, or data conversions.  13c Describe any methods used to tabulate or visually display results of individual studies and syntheses  13d Describe any methods used to synthesise results and provide a rationale for the choice(s). If meta-analysis was performed, describe the model(s), method(s) to identify the presence and extent of statistical heterogeneity, and software package(s) used  13e Describe any methods used to explore possible causes of heterogeneity among study results (e.g. subgroup analysis, meta-regression)  13f Describe any sensitivity analyses conducted to assess robustness of the synthesised results   1. Describe any methods used to assess risk of bias due to missing results in a synthesis (arising from reporting biases) 2. Describe any methods used to assess certainty (or confidence) in the body of evidence for an outcome. | 6, 7  6  6  6, 7  7  -  8  7  7  6,7  -  8  -  -  -  -  - |
| Results  Study selection  Study characteristics  Risk of bias in studies  Results of individual studies  Results of syntheses  Reporting biases  Certainty of evidence |  | 16a Describe the results of the search and selection process, from the number of records identified in the search to the number of studies included in the review, ideally using a flow diagram  16b Cite studies that might appear to meet the inclusion criteria, but which were excluded, and explain why they were excluded   1. Cite each included study and present its characteristics 2. Present assessments of risk of bias for each included study 3. For all outcomes, present, for each study: (a) summary statistics for each group (where appropriate) and (b) an effect estimate and its precision (e.g. confidence/credible interval), ideally using structured tables or plots.   20a.For each synthesis, briefly summarise the characteristics and risk of bias among contributing studies.  20b. Present results of all statistical syntheses conducted. If meta-analysis was done, present for each the summary estimate and its precision (e.g. confidence/credible interval) and measures of statistical heterogeneity. If comparing groups, describe the direction of the effect  20c. Present results of all investigations of possible causes of heterogeneity among study results.  20d. Present results of all sensitivity analyses conducted to assess the robustness of the synthesised results.  21. Present assessments of risk of bias due to missing results (arising from reporting biases) for each synthesis assessed  22. Present assessments of certainty (or confidence) in the body of evidence for each outcome assessed. | 6,7  -  8  -  -  -  -  -  -  -  - |
| **Discussion**  Discussion |  | 23a Provide a general interpretation of the results in the context of other evidence  23b Discuss any limitations of the evidence included in the review.  23c Discuss any limitations of the review processes used.  23 dDiscuss implications of the results for practice, policy, and future research | 10, 11, 12  13  13  13 |
| **Other information**  **Support** |  | 24a Provide registration information for the review, including register name and registration number, or state that the review was not registered  24b Indicate where the review protocol can be accessed, or state that a protocol was not prepared  24c Describe and explain any amendments to information provided at registration or in the protocol  25. Describe sources of financial or non-financial support for the review, and the role of the funders or sponsors in the review. | 6  -  -  14 |
| Competing interest  Availability of data, code, and other materials |  | 26. Declare any competing interests of review authors  27. Report which of the following are publicly available and where they can be found: template data collection forms; data extracted from included studies; data used for all analyses; analytic code; any other materials used in the review. | 14  - |

*From:* Page, M. J., McKenzie, J. E., Bossuyt, P. M., Boutron, I., Hoffman, T. C., Mulrow, C. D., Shamseer, L., Tetzlaff, J. M., Akl, E. A., Brennan, S. E., Chou, R., Glanville, J., Grimshaw, J. M., Hróbjartsson, A., Lalu, M. M., Li, T., Loder, E. W., Mayo-Wilson, E., McDonald, S., McGuinness, L. A., & Moher, D. (2021). The PRISMA 2020 statement: An updated guideline for reporting systematic reviews. *International Journal of Surgery, 88*, 1-9. <https://doi.org/10.1016/j.ijsu.2021.105906>
